# Impact of natural and socio-economic factors on varicella incidence in children in Shanghai, 2013-2022

**DOI:** 10.3389/fpubh.2025.1565717

**Published:** 2025-05-27

**Authors:** Chunmei Duan, Yan Zhang, Qian Zhang, Shuzhi Zhang, Peisong Zhong, Gang Gong, Yefan Zhu, Jie Fei, Jingjing Zhao, Yanling Sun, Yaqing Jin, Yunjie Ren, Yihan Lu, Ye Yao, Hongjie Yu

**Affiliations:** ^1^Jiading District Center for Disease Control and Prevention, Shanghai, China; ^2^School of Public Health, Fudan University, Shanghai, China; ^3^The Key Laboratory of Public Health Safety of Ministry of Education, Fudan University, Shanghai, China

**Keywords:** varicella, incidence, children, vaccination, natural factors, socio-economic factors

## Abstract

**Background:**

Since varicella is already known to be a globally distributed disease, the focus should be more on its transmissibility or disease burden. The incidence of varicella is affected by natural and socio-economic factors. However, it is unclear how these factors synergetically impact the dynamics of varicella transmission and control.

**Methods:**

We conducted a retrospective analysis of varicella cases in children aged 0–17 years from 2013 to 2022 in Jiading District, Shanghai, China. First, we evaluated demographic characteristics, epidemiological trends of varicella. And then, we explored the impact of two-dose varicella vaccine (VarV) program on varicella incidence using interrupted time-series analyses, and assessed the influence of natural and socio-economic factors using principal component analysis and multivariate regression. Spatial analysis was conducted to compare varicella epidemiology.

**Results:**

Our analysis includes 6,482 reported varicella cases, with a higher incidence observed among males (58.67%). Regional differences were noted, with the highest incidence in the western region and the lowest in the central region. Before the implementation of the two-dose VarV program, varicella incidence increased by 0.28 cases per 100,000 per month. Following the two-dose VarV program’s introduction, the incidence rate decreased by 0.49 cases per 100,000 per month, with an impressive 79.10% reduction in the annual average incidence among children aged 4–6 years. By analyzing the impact of demographic characteristics, healthcare capacity, economic level, air pollutants, and meteorological factors on the incidence of varicella, we found that the child population ratio and VarV program were most strongly associated with varicella incidence.

**Conclusion:**

The study underscores the importance of sustained monitoring of child population ratio and VarV program to reduce varicella transmission and protect vulnerable groups.

## 1 Introduction

Varicella, also known as chickenpox, is an acute febrile infectious disease caused by the varicella zoster virus (VZV) (1). Its main symptom is a characteristic rash, often accompanied by fever and other symptoms (2). It shows a predominance in the winter/spring or cool/dry month and is primarily transmitted through airborne droplets, aerosols, and direct contact (3, 4). Though varicella is generally a benign, self-limiting disease in children (1, 5), it can occasionally lead to serious complications such as encephalitis, pneumonia, or sepsis, requiring hospitalization or even resulting in death (6). The global distribution of varicella hightlights its high infectiousness and substantial disease burden it imposes on families and society (7). In China, varicella remains a major public health challenge, with 11,990 outbreaks involving 354,082 cases reported between 2006 and 2022 (8). As with many other infectious diseases, active immunization serves as the primary preventative measure against varicella. Recent studies have indicated that the protective effect of a single dose of the varicella vaccine (VarV) is limited, with breakthrough infection rate ranging from 0.12 to 2.5% in China (9), 9.5% in the United States (10), 12.7% in Germany (11). During varicella outbreaks, 56.5–83.3% of cases had a history of one dose VarV vaccination before disease onset (12–14). In contrast, the two-dose VarV regimen provided nearly 95% protection against all grades of severity of varicella (6), significantly reducing breakthrough cases compared to the one-dose regimen. Despite the substantial scientific evidence supporting the benefits of varicella vaccination, VarV has not been widely included in routine immunization programs in most regions of China (8). Barriers to vaccine adoption include cost, inconsistent health policies, and public acceptance issues. In a budgetary analysis for the cohort of newborns in 2023, Zhang H et al. (15) estimated that the projected aggregate government costs were $221.64 million for varicella vaccine. Taking Shanghai as an example, the domestic varicella vaccine is priced at ¥141.5–166 (20.51–24.06 USD) per dose (16, 17), while the imported vaccine costs ¥330 (47.83 USD) per dose. All conversions use 2019 average rates (1 USD = 6.90 CNY) (18). These cost remains a significant concern for many families, especially in regions with lower economic development (5, 15). Inconsistent health policies across different provinces and municipalities (19–23) have hindered the widespread implementation of varicella vaccination programs. Public acceptance of the vaccine is also a challenge, as some parents may be reluctant to vaccinate their children due to perceiving varicella vaccination as unnecessary, concerns regarding side effects, or doubts the vaccine’s effectiveness (5, 24, 25).

Besides, previous studies have identified meteorologic conditions (26–30), air pollution (31, 32), demographic and socio-economic factors (33, 34) as key influences of varicella incidence. Suzuki et al. (26) found that temperature and the school term interact to affect varicella transmission in Japan. Zhang et al. (30) discovered significant nonlinear effects of meteorological factors like temperature, air pressure, diurnal temperature range, and sunshine hours on varicella incidence. Air pollutants, particularly particulate matter 10 μm or less (PM10) and NO_2_, has also been linked to varicella risk (31, 32). Demographic and socioeconomic factors, including age and income, have been shown to influence VZV seroprevalence (33). However, the synergistic impact of these natural and socio-economic factors on varicella transmission and control remains unclear, especially in complex urban settings.

Jiading District, located in the northwestern suburbs of Shanghai, serves as an important urban and industrial hub, characterized by a diverse population and a blend of urban and suburban environments. In 2022, the floating population accounted for 54.92% of the resident population (35). The region has a typical subtropical monsoon climate with significant seasonal changes, offering a natural setting to study the effects of meteorological factors on disease transmission. Comprehensive health, demographic, and environmental data are accessible through local health departments, the Public Security Bureau, and statistical yearbooks, supporting robust epidemiological analysis. Jiading’s public health significance is underscored by its substantial childhood population (36) and considerable varicella disease burden (37). Furthermore, socio-economic diversity of Jiading District and uneven distribution of healthcare resources provide an opportunity to examine how these factors influence varicella incidence and vaccination coverage. Overall, Jiading District’s unique environmental, demographic, and socio-economic characteristics made it an ideal setting for investigating the multifaceted drivers of varicella incidence among children. In this study, we investigated the varicella incidence rates in children in Jiading District, Shanghai from 2013 to 2022 and explored the impact of immunization programs, natural and socio-economic factors on variella epidemiology. By integrating data on demographics, healthcare capacity, economic conditions, air pollution, and meteorological factors over a decade, this study comprehensively analyses their interplay in varicella incidence. Using advanced statistical methods, including principal component analysis, multiple regression, and spatial analysis, we identify key determinants and their interactions, highlighting the importance of a multi-faceted approach to disease control.

The anticipated findings are expected to provide actionable insights for public health policy and varicella control strategies. They will contribute to optimizing vaccination programs by identifying high-risk populations and geographic areas, guiding targeted interventions to address socio-economic disparities, and informing the development of predictive models for forecasting varicella outbreaks based on environmental and socio-economic indicators. Furthermore, as a major urban and industrial hub in Shanghai, Jiading District offers a valuable case study whose insights may be applicable to other urban settings, thereby enhancing our broader understanding of varicella transmission dynamics.

## 2 Materials and methods

### 2.1 Study setting

Jiading District, located in the center of the Yangtze River Delta region. It covers a 464-square-kilometer area to the northwest of Shanghai Municipality, China. Its central coordinates are 121°26′E and 31°39’N, with a resident population of 1.86 million, which account for 7.66% of the Shanghai population. Jiading District consists of four regions in the spatial pattern: central (Jiading Town Street, Xincheng Road Street, Juyuan New Area, and Malu Town), western (Anting Town), southern (Zhenxin Street, Nanxiang Town, and Jiangqiao Town) and northern (Jiading Industrial Zone, Waigang Town, Xuhang Town, and Huating Town) (38). The four regions are core functional area, automotive research and development industrial area, urban functional area and ecological science and innovation area of Jiading district, respectively.

Since 2004, Shanghai has included varicella in its monitoring and reporting of infectious diseases, aiming to track the varicella burden and epidemiological changes within the city. Local medical institutions conducted direct reporting and online management once health providers and doctors diagnose patients with varicella. Since January 1, 2018, varicella has been managed as a Class C infectious disease in Shanghai. All varicella cases should be reported within 24 h through the Infectious Disease Epidemic Surveillance Information System. The report included name, sex, age, occupation, current home address, date of onset, date of diagnosis, name of disease, reporting unit, etc.

Since 1999, Shanghai has introduced VarV as class II vaccine and provided one dose of vaccination on a voluntary basis for healthy children over 1 year old. The VarV in Shanghai was vaccinated two doses instead of one dose since November 7, 2017. Children should receive first dose of VarV at 12 months old and a second dose at 4 years of age. Since August 1, 2018, VarV has been included in the Shanghai immunization program, and the municipal government has provided free vaccination for eligible children on or after August 1, 2014. Other citizens in Shanghai have the option to receive the varicella vaccine voluntarily and at their own expense based on their individual needs.

### 2.2 Data sources

The data of varicella cases from January 1, 2013 to December 31, 2022 were collected from the infectious disease reporting information management system of China Information System for Disease Control and Prevention (CISDCP) according to the date of onset and the patient’s current home address. The vaccination information was collected from the vaccination records of residents in Jiading District, Shanghai. Vaccination data and case information were matched by name, personal identity number, date of birth, and then anonymized. Discrepancies were resolved through cross-referencing and verification with healthcare facilities. Cases with incomplete or unverifiable data were excluded, and minor inconsistencies were addressed through data imputation. Standardized reporting protocols and regular audits further ensured data integrity. The population data by age groups were obtained from the Jiading District Public Security Bureau of Shanghai.

The natural and socio-economic factors including demographic characteristics, healthcare capacity, economic level, air pollutants and meteorological factors of 12 street and town under the jurisdiction were counted by year. Children population, sex ratio, household registration ratio, child population density were used as indicators of demographic characteristics. VarV immunization program, secondary and above comprehensive medical institutions, total number of patients, number of health check-ups were used as indicators of healthcare capacity. Completed investment in fixed assets, industrial output value above the set scale, profits of enterprises above the set scale, comprehensive energy consumption, unit output energy consumption were used as indicators of economic level. Particles with an aerodynamic diameter less than 10 μm (PM10), sulfur dioxide (SO_2_), nitrogen dioxide (NO_2_), acid rain frequency were used as air pollutants. Annual average temperature, annual rainfall, sunshine duration, relative humidity were used as meteorological factors. VarV immunization program was a categorical variable with a value of 0 for one-dose immunization program and 1 for two-dose immunization program, respectively. Continuous variables of healthcare capacity, economic level, air pollutants and meteorological factors were obtained from Jiading Statistical Yearbook (39).

### 2.3 Statistical analysis

Annual and monthly varicella incidence rates were calculated by dividing the respective case numbers by the average annual child population and then scaling up by 100,000 to express the rate per 100,000. Based on the vaccination ages for the first and second doses of the varicella vaccine and considering data comparability, we categorized all children aged 0–17 years (40) into six age groups: 0 years, 1–3 years, 4–6 years, 5–7 years, 9–12 years, and 13–17 years.

Normality was assessed by using the Shapiro–Wilk test. Continuous variables conforming to normal distribution were reported as mean ± standard deviation (SD), while those not conforming to normal distribution were presented as median and inter-quartile range (IQR). Categorical variables were expressed as numbers (percentage). Group comparisons were conducted using either a two-sided Chi-square test for categorical variables or a Wilcoxon rank sum test for continuous variables that did not meet normality assumptions.

Interrupted time-series analysis was conducted to evaluate changes in varicella incidence trends following the implementation of the two-dose VarV program in Shanghai. An additive model was employed to account for seasonal variations, while an auto-regressive moving average (ARMA) term addressed residual autocorrelation. Based on specific policy change (41, 42), actual vaccination data (Supplementary Figure S1), and published literature (22), November and December 2017 were designated as a transition period during which the two-dose VarV program was recommended. To assess the influence of sex and age on varicella incidence, we performed stratified analyses by sex (male and female) and age groups (1–3 years and 4–6 years). These age strata correspond to the primary target populations for the one-dose and two-dose varicella vaccine programs, respectively. The validity of the segmented regression model was assessed through visual inspection of correlograms and residual analysis to ensure model adequacy and adherence to statistical assumptions.

The relationship between each variables was examined using cross-correlation Spearman rank correlation analyses. Variance inflation factors (VIF) was performed to estimate the multicollinearity of the independent variables. Partial correlation coefficient squared (Partial R^2^) was used to gages the contribution of a particular independent variable to a model’s prediction after controlling for all other variables. Principal component analysis (PCA) was employed to reduce the dimensionality of independent variables. Raw data were standardized to remove dimensional differences between indicators. Prior to PCA, the Kaiser-Meyer-Olkin (KMO) measure of sampling adequacy and Bartlett’s test of sphericity were conducted to ensure data adequacy. Principal components were extracted based on eigenvalues and cumulative variance contribution rates, with selection of the first n components explaining 80% of the total variance. Multiple regression analysis was then used to examine the relationship between annual varicella incidence and these principal components, accounting for interaction terms between air pollution and meteorological factors.

Spatial distribution maps were conducted in ArcGIS 10.8.1 (Esri., United States of America.) to compare pre- and post-intervention data, including annual average incidence, household registration ratio, child population density, and total number of patients.

All statistical analyses were conducted using R version 4.4.0 from The R Project for Statistical Computing, Shanghai. Two-tailed tests were employed, and statistical significance was defined as *p* value < 0.05.

## 3 Results

### 3.1 Characteristics of varicella cases

From 2013 to 2022, a total of 6,482 varicella cases in individuals aged 0–17 years were reported in Jiading District (Supplementary Table S1). Among these cases, 3,803 (58.67%) were male and 2,679 (41.33%) were female, median age (IQR), 6 (3-11) years. Compared to the one-dose VarV program period, the median age of varicella cases increased from 6 years to 7 years during two-dose VarV program period (*p* = 0.002). The highest number of varicella cases occurred in children aged 4–6 years (25.08%), followed by those aged 7–9 years (17.88%). However, during two-dose VarV program period, the peak of varicella cases shifted to the 1–3 years age group (21.81%). More than half (51.23%) of the cases were reported in students. Varicella cases in the southern and central regions accounted for 32.38% and 27.06% of the total, respectively. More importantly, the number of varicella cases sharply decreased after children received two doses of the VarV.

### 3.2 Impact of VarV program on the incidence of varicella

From 2013 to 2022, the average annual incidence of varicella in Jiading District was 381.26 cases per 100,000. From 2013 to 2017, the annual incidence of varicella in children showed an overall upward trend, increased from 498.62 cases per 100,000 to 730.46 cases per 100,000. From 2018 to 2022, the incidence of varicella in children showed an overall downward trend, which decreased from 410.11 cases per 100,000 to 115.60 cases per 100,000. There was a statistically significant difference in the incidence rate among 2013 to 2022 (χ2 = 1515.67, *p* < 0.001) (Supplementary Figure S2).

The average annual incidence of varicella in children among one-dose VarV program period was 550.53 cases per 100,000. The incidence of male (591.04 cases per 100,000) was higher than that of female (502.00 cases per 100,000), and the incidence of children aged less than 1 year and 4–6 years was higher than those of other age groups. Regional differences were noted, the highest incidence of varicella was observed in the western region of Jiading District (682.61 cases per 100,000). In contrast, the central region had the lowest incidence (488.20 cases per 100,000). During two-dose VarV program period, the annual average incidence of varicella in children decreased to 241.92 cases per 100,000. Compared with the period of one-dose VarV program period, the annual average incidence of varicella in children aged 4–6 years decreased the most by 79.10%, followed by 7–9 years group with a decrease of 61.73%. The lowest incidence was observed in the central region (197.26 cases per 100,000). Overall, the incidence of varicella in children decreased by more than 50% in all four districts of Jiading District compared to one-dose Varv program period. The related data were shown in Table 1.

**Table 1:** Annual average incidence of varicella in children in Jiading District, Shanghai, 2013–2022.

Characteristics	Annual average incidence (per 100,000)			Percentage of decrease in incidence (%)*
	All study period	One-dose VarV program period	Two-dose VarV program period	
Sex				
Male	414.78	591.04	266.83	54.85
Female	342.03	502.00	213.33	57.50
Age group				
<1 year	1019.40	1453.86	697.89	52.00
1–3 years	344.07	468.78	260.64	44.40
4–6 years	419.61	745.74	155.89	79.10
7–9 years	354.76	529.05	202.47	61.73
10–12 years	399.01	520.07	292.38	43.78
13–17 years	288.95	335.40	246.26	26.58
Region				
Central	320.02	488.20	197.26	59.59
Western	446.79	682.61	268.77	60.63
Southern	395.92	552.63	272.64	50.67
Northern	403.53	540.93	246.95	54.35
Overall	381.26	550.53	241.92	56.06

*Comparison of the proportion of children experiencing reduced varicella incidence between the two-dose and one-dose VarV programs. VarV, varicella vaccine.

### 3.3 Association of two-dose VarV program with the monthly incidence of varicella

**Figure 1:**
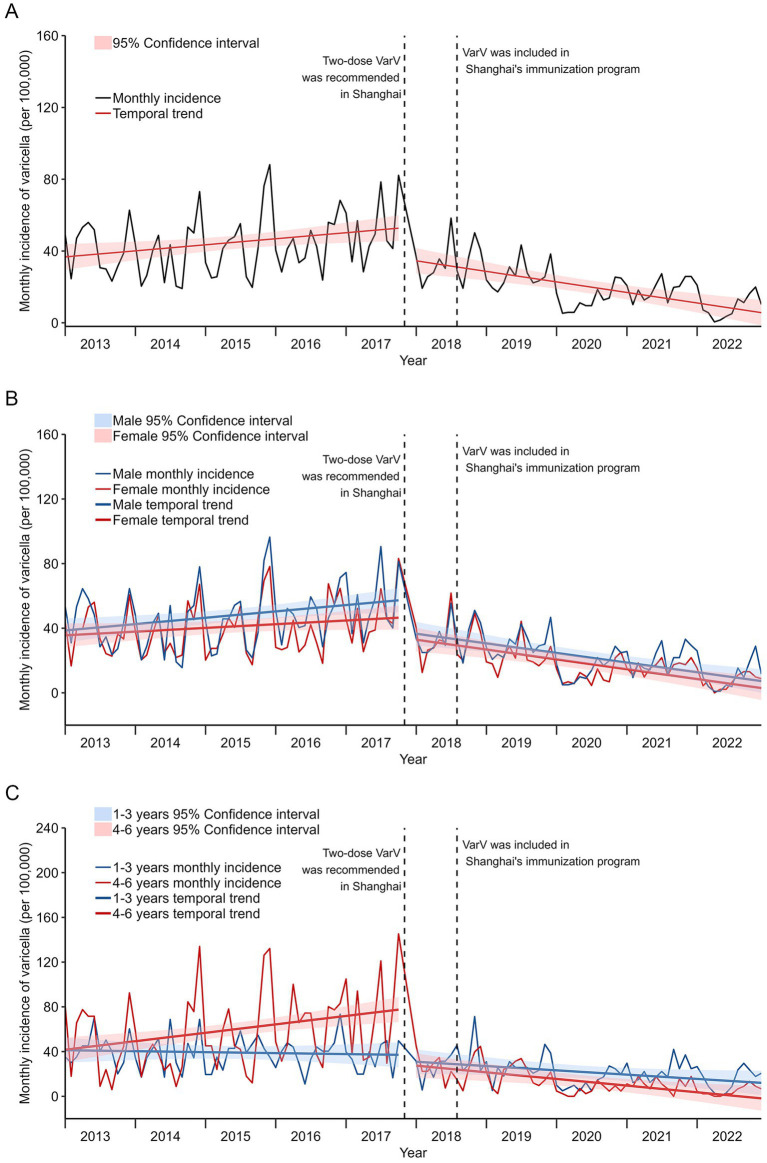
Association of the implementation of the two-dose VarV program with the monthly incidence of varicella in children in Jiading District, 2013–2022. **(A)** All cases (unstratified); **(B)** Stratified by sex; **(C)** Stratified by age groups. The black line represents the actual monthly varicella incidence in Jiading district. The red slope lines are incidence trends estimated via segmented regression. The red shading areas are the 95% CI estimated via segmented regression. The dotted vertical lines represent the two-dose VarV was recommended in Shanghai (November 2017) and VarV was included in immunization program of Shanghai (August 2018).

Before the recommendation of the two-dose VarV vaccine in Shanghai, the monthly incidence of varicella was 36.73 cases per 100,000 in January 2013. There was an increase of 0.28 cases per 100,000 per month (*t* = 2.55, *p* = 0.012, 95% CI: 0.06–0.50), reaching 82.19 cases per 100,000 in October 2017. After the implementation of the two-dose VarV vaccine, a significant reduction in the monthly incidence trend was observed (slope: −0.49 per 100,000 per month, *t* = −4.98, *p* < 0.001), starting from January 2018 (Figure 1A). No statistically significant difference was observed in the changes of varicella incidence between males and females following the implementation of the two-dose VarV program (Pre-vaccination: *t* = −0.84, *p* = 0.401. Post-vaccination: *t* = 0.56, *p* = 0.577) (Figure 1B). A declining trend in varicella incidence of 1–3 years was noted both before and after the two-dose VarV implementation, though the difference did not reach statistical significance (*t* = −1.04, *p* = 0.300). Prior to the two-dose VarV implementated, children aged 4–6 years exhibited an upward trend of 0.63 cases per 100,000 population per month in varicella incidence (*t* = 2.91, *p* = 0.004), which reversed to a statistically significant downward trend of 1.12 cases per 100,000 population per month post-implementation (*t* = −2.62, *p* ≤ 0.001) (Figure 1C).

### 3.4 Impact of natural and socio-economic factors on the incidence of varicella

**Figure 2:**
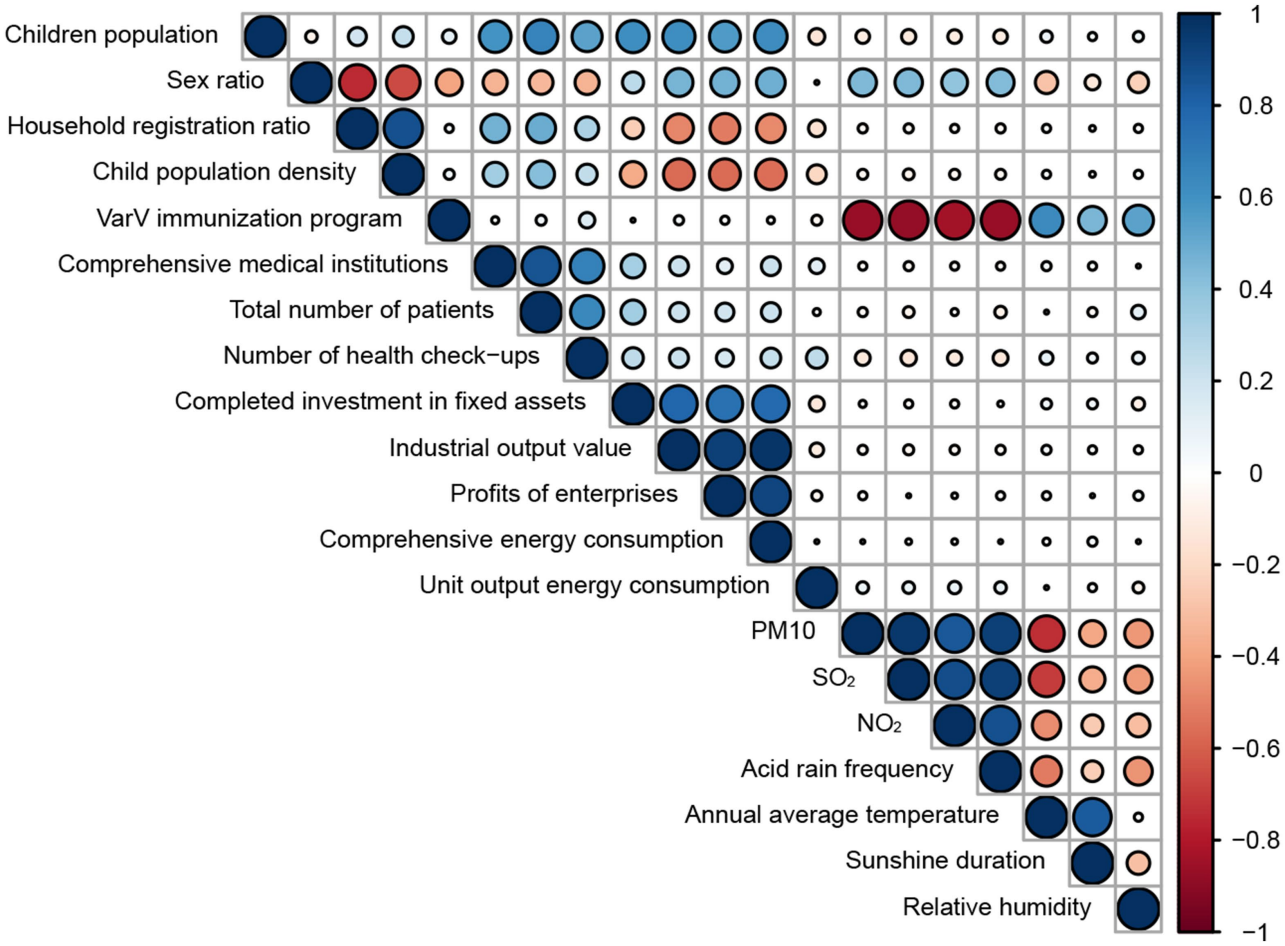
Result of Cross-correlation Spearman rank correlation of natural and socio-economic influencing factors.

In our study, 20 variables representing natural and socio-economic factors were included in the analysis. Cross-correlation Spearman rank correlation showed that children population was positive correlation with total number of patients, completed investment in fixed assets, industrial output value, comprehensive energy consumption, sex ratio was negative correlation with household registration ratio, child population density, VarV immunization program was negative correlation with PM10, SO_2_, NO_2_ and acid rain frequency, positive correlation with annual average temperature, annual average temperature was negative correlation with PM10, SO_2_ and positive correlation with sunshine duration (Figure 2). The results of the multicollinearity test showed that the VIF of 10 variables was greater than 10, which inferred that there was a serious multicollinearity between the variables (Supplementary Table S2). In view of this phenomenon, the dimensionality reduction of variables was carried out in our study.

We performed principal component analysis in four categories: demographic characteristics, healthcare capacity, economic level, environmental factors, respectively. The KOM of each category was 0.69, 0.66, 0.64, 0.61, respectively. Bartlett’s tests of sphericity were statistically significant (*p* < 0.001).

The analyses revealed the following key findings regarding the principal component extraction and their variance contributions.

**Figure 3:**
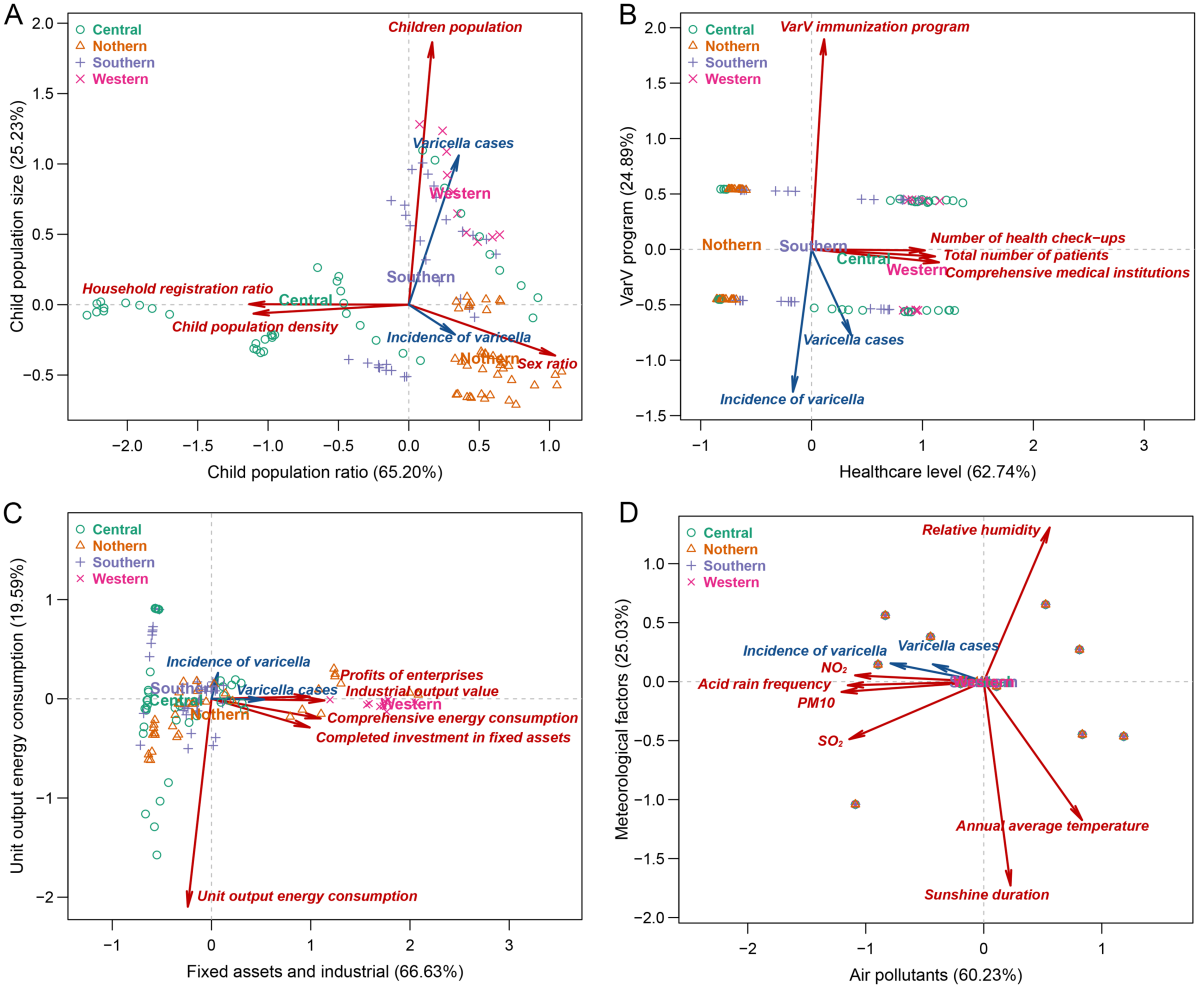
Principal component analysis of natural and socio-economic influencing factors on varicella incidence in 12 street and towns of Jiading District from 2013 to 2022. **(A)** Demographic Characteristics; **(B)** Healthcare Capacity; **(C)** Economic Level; **(D)** Air Pollutants and Meteorological Factors. All statistical variables have been standardized. The plot utilizes four distinct colors to denote the regional affiliations of each case. Variables are depicted as arrows indicating increasing values, with the origin (point [0,0]) marking the mean of all variables. When two variables share the same directional orientation, it suggest a positive correlation between them. In contrast, variables pointing in opposite directions imply a negative correlation. Red arrows represent natural and socio-economic influencing factors. Additionally, two supplementary variables, varicella incidence and varicella cases, are introduced, signified by dark blue arrows.

Demographic characteristics (Figure 3A): Two principal components accounted for 90.43% of the variance, with the first principal component (PC1) explaining 65.20% and the second principal component (PC2) explaining 25.23% of the total variance. The household registration ratio had the most significant positive contribution to PC1, followed by child population density (negative effect) and sex ratio (positive effect). Consequently, PC1 could be termed the “Child Population Ratio.” The child population was the most substantial contributor to PC2, thus PC2 could be referred to as the “Child Population Size.” In the PCA biplot, two additional variables, varicella incidence and varicella cases, were included. Varicella incidence was significantly correlated with PC1 (*p* = 0.002), while varicella cases were significantly associated with both PC1 and PC2 (both *p* < 0.001). The varicella incidence was found to align with the sex ratio and be inversely related to household registration ratio and population density in the context of PC1.

Healthcare capacity (Figure 3B): Two principal components explained 87.63% of the variance. PC1, which could be labeled as “Healthcare Level” was positively influenced by the number of secondary and above comprehensive medical institutions, total patient count, and the number of health check-ups. The VarV immunization program of Shanghai contributed most significantly to PC2, also with a negative effect. Both varicella incidence and varicella cases were significantly associated with PC1 and PC2 (*p* < 0.05), and they were significantly explained by both PC1 and PC2 (*p* < 0.05), showing an opposition to the VarV immunization program of Shanghai.

Economic level (Figure 3C): Two principal components were extracted, accounting for 86.22% of the variance. PC1 combined factors of fixed asset investment (completed investment in fixed assets) and industrial economic indicators (industrial output value above the designated scale, profits of enterprises above the designated scale, and comprehensive energy consumption), influencing PC1 positively. Unit output energy consumption was the primary driver of PC2 with a negative effect, exerting a negative correlation on varicella incidence.

Environmental factors (Figure 3D): Two principal components were identified, accounting for 85.26% of the variance. Air pollutants (PM10, SO_2_, NO_2_, acid rain frequency) significantly contributed to PC1 with a negative influence, while meteorological factors (sunshine duration, relative humidity) substantially contributed to PC2. Both varicella incidence and varicella cases were significantly associated with PC1 (*p* < 0.001), with no significant difference in PC2. Both varicella incidence and varicella cases were significantly explained by PC1 (*p* < 0.001) and were consistent with air pollutants. The arrow representing varicella incidence was in the opposite direction to meteorological factors but showed no significant correlation with PC2 (*p* = 0.248).

Based on eigenvalues and cumulative variance contribution rates, 8 components, including child population ratio, child population size, healthcare level, VarV program, fixed assets and industrial, unit output energy consumption, air Pollutants, and Meteorological Factors, were retained.

The results of multiple regression (*F* = 15.67, *p* < 0.001) indicated that principal component of child population ratio (coefficient = 60.03, *p* = 0.021) have positive impact on the annual incidence of varicella among children, while VarV Program of Shanghai has a negative impact (coefficient = −229.40, *p* = 0.001) on the annual incidence of varicella among children, the differences were all statistically significant. Other principal components, including child population size, healthcare level, fixed assets and industrial output, unit output energy consumption, air pollutants, and meteorological factors, did not exhibit statistically significant associations with annual varicella incidence (Table 2). Further analysis of natural and socio-economic factors showed that the household registration ratio demonstrated a significant negative correlation (coefficient = −1.34, *p* = 0.001), and child population density showed a significant positive correlation (coefficient = 0.11, *p* = 0.038) with annual varicella incidence. However, the sex ratio did not achieve statistical significance (coefficient = −8.40, *p* = 0.121). The VarV Program continued to show a strong negative association with incidence (coefficient = −288.27, p = 0.001). No significant interactions were found between air pollutants and meteorological factors on varicella incidence (*p* = 0.126) (Table 3).

**Table 2:** Associations between principal components and varicella incidence in children in Jiading District, Shanghai, 2013–2022.

Variables	Coefficient	Standard error	*t* value	*p* value	95% CI
Constant	386.61	14.24	27.15	<0.001**	358.39 ~ 414.82
Child population ratio	60.03	25.62	2.34	0.021*	9.26 ~ 110.79
Child population size	28.25	48.65	0.58	0.563	−68.15 ~ 124.66
Healthcare level	−13.20	32.92	−0.40	0.689	−78.44 ~ 52.04
VarV program	−229.40	64.43	−3.56	0.001*	−357.07 ~ −101.74
Fixed assets and industrial	−3.95	23.72	−0.17	0.868	−50.96 ~ 43.05
Unit output energy consumption	56.10	39.80	1.41	0.161	−22.76 ~ 134.96
Air pollutants	−43.69	42.18	−1.04	0.303	−127.28 ~ 39.9
Meteorological factors	32.14	28.70	1.12	0.265	−24.73 ~ 89.01

VarV, varicella vaccine; CI, Confidence interval. *, *p* < 0.05; **, *p* < 0.001.

**Table 3:** Impact of natural and socio-economic factors on varicella incidence in children in Shanghai, 2013–2022.

Variables	Coefficient	Standard error	*t* value	*p* value	95% CI
Constant	1465.07	661.80	2.214	0.029*	153.28 ~ 2776.87
Children population (100,000 persons)	−40.98	287.93	−0.14	0.887	−611.7 ~ 529.74
Sex ratio (male to female, %)	−8.40	5.38	−1.56	0.121	−19.07 ~ 2.27
Household registration ratio (registered to non-registered, %)	−1.34	0.40	−3.39	0.001**	−2.13 ~ −0.56
Child population density (person/square-kilometer)	0.11	0.05	2.10	0.038*	0.01 ~ 0.2
Healthcare level	−4.73	32.02	−0.15	0.883	−68.21 ~ 58.75
VarV program	−288.27	67.74	−4.26	<0.001**	−422.54 ~ −153.99
Fixed assets and industrial	26.63	24.87	1.07	0.287	−22.66 ~ 75.92
Unit output energy consumption	31.26	41.78	0.75	0.456	−51.56 ~ 114.07
Air pollutants	−46.96	49.75	−0.94	0.347	−145.57 ~ 51.65
Meteorological factors	44.50	30.43	1.46	0.147	−15.81 ~ 104.82
Air pollutants: meteorological factors	58.12	37.17	1.56	0.121	−15.55 ~ 131.79

VarV, varicella vaccine; CI, Confidence interval. *, *p* < 0.05; **, *p* < 0.001.

### 3.5 Spatial distribution of varicella incidence, demographic and health service indicators

**Figure 4:**
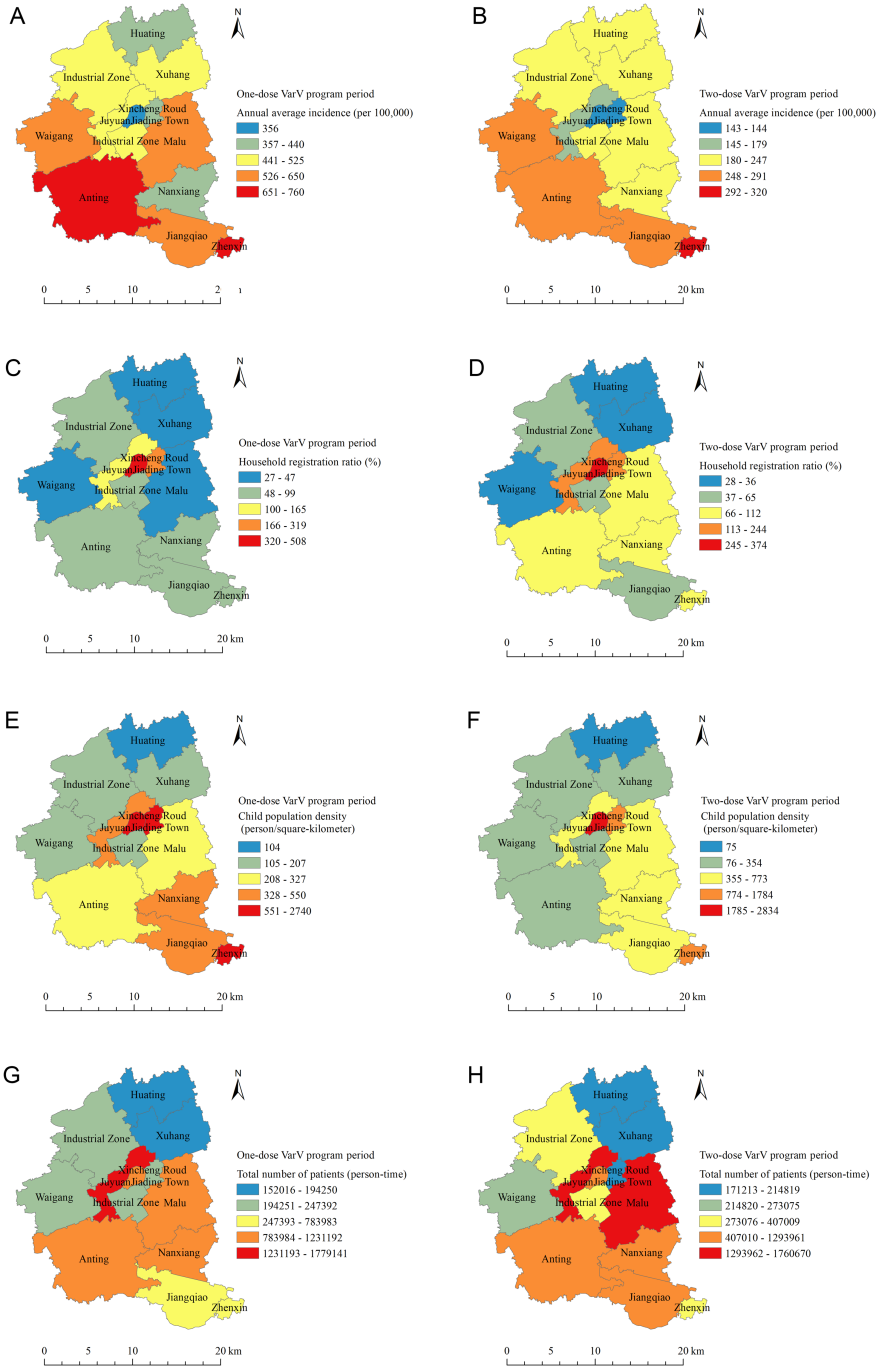
Spatial distribution of varicella incidence, Demographic and Health Service Indicators. **(A)** Annual average incidence (per 100,000) in one-dose VarV program period. **(B)** Annual average incidence (per 100,000) in two-dose VarV program period. **(C)** Household registration ratio (registered to non-registered, %) in one-dose VarV program period. **(D)** Household registration ratio (registered to non-registered, %) in two-dose VarV program period. **(E)** Child population density (person/square-kilometer) in one-dose VarV program period. **(F)** Child population density (person/square-kilometer) in two-dose VarV program period. **(G)** Annual average total number of patients (person-time) in one-dose VarV program period. **(H)** Annual average total number of patients (person-time) in two-dose VarV program period.

The spatial analysis revealed significant regional disparities in varicella incidence rates. The western region of Jiading District, Anting Town, experienced high incidence rates during the one-dose VarV program period, with a notable decrease following the introduction of the two-dose VarV program. In contrast, Jiading Town Street and Juyuan New Area in the central regions maintained consistently low incidence rates. The southern region showed moderate to high incidence rates, with Zhenxin Street exhibiting high rates throughout the study period, while Jiangqiao Town and Nanxiang Town showed variability. Northern regions generally reported low to moderate incidence rates (Figures 4A,B). In central regions, Jiading Town and Xincheng Road Street had higher ratios of registered to non-registered children, higher child population density, Juyuan New Area and Malu Town had greater total number of patients. Northern regions had lower ratios of registered children, lower population density, and fewer outpatient visits. Southern regions displayed moderate levels of household registration ratio but moderate to high population density and total number of patients. Anting Town showed low to moderate levels of household registration ratio and high population density, but higher total number of patients (Figures 4C–H).

## 4 Discussion

The present study investigated varicella incidence trends and the impact of natural and socio-economic factors on varicella dynamics in Jiading, Shanghai. Initially, there was a concerning rise in varicella cases among children, peaking in 2017. However, following the introduction of the two-dose VarV vaccination program in November 2017, a significant decline in incidence rates was observed from 2018 onwards. The average annual incidence of varicella in children decreased by 56.06%, from 550.53 cases per 100,000 during the one-dose VarV period to 241.92 cases per 100,000 during the two-dose VarV period. Additionally, the monthly incidence trend reversed, showing a significant decline of −0.49 cases per 100,000 per month (*p* < 0.001) during the two-dose VarV period, compared to the upward trend of 0.28 cases per 100,000 per month (*p* = 0.012) during the one-dose VarV period. This decline underscores the effectiveness of the vaccination program in reducing the disease burden. The results of principal component analysis and multiple linear regression confirmed that the implementation of the two-dose VarV program significantly reduced children varicella cases, consistent with findings from studies in the United States (43), Beijing (44), Shanghai (22, 45), and Wuxi (20), China. In additional, while public health and social measures were implemented to control the COVID-19 pandemic, their influence on the transmission dynamics of varicella was found to be relatively limited (22, 46).

The introduction of the two-dose VarV program led to a reduction in varicella incidence across all age groups of children, highlighting the program’s herd immunity effects (47, 48). This pattern aligns with observations in the United States, where a substantial decline in incidence was noted for all age groups (49). High vaccine coverage was identified as critical to reducing varicella incidence (50), with a 20% increase in coverage leading to a 27–31% reduction in cases (51). Notably, Shanghai’s two-dose VarV coverage rate was higher than in other regions of China (19, 22, 44), which may have contributed to a greater reduction in varicella incidence. The most significant reduction occurred in children aged 4–6 years, with a 79.10% decrease in incidence. The most significant reduction occurred in children aged 4–6 years, with a 79.10% decrease in incidence. It demonstrated that the second dose of the varicella vaccine is specifically administered to individuals aged 4 years and above, with children aged 4–6 years being the primary recipients. This targeted vaccination strategy has resulted in enhanced immune protection for this age group (45). Consequently, there has been a significant reduction in breakthrough cases (6), leding to a marked decrease in the overall incidence of varicella among children aged 4–6 years. As the proportion of varicella cases among children aged 4–6 years has decreased due to the effective vaccination program, the proportion of cases in the 0 year, 1–3 years, 10–12 years, and 13–17 years groups has correspondingly increased. Notably, children aged 1–3 years are at a higher risk of contracting varicella. This increased susceptibility is attributed to their still-developing immune systems, which are not as robust as those of older children. Therefore, under the same conditions, children aged 1–3 years are more susceptible to varicella compared to children in other age groups.

The spatial analysis revealed distinct regional patterns in varicella incidence, with the highest rates in western area of Jiading District and the lowest in central areas. While these disparities initially appeared correlated with urban–rural gradients, further examination of underlying spatial determinants proved insightful. The western region’s elevated incidence likely results from synergistic factors: its border location adjacent to Jiangsu Province facilitates cross-regional transmission, while lower vaccination coverage (52) in these transitional zones may compound risk. Central areas benefited not only from higher population immunity due to better healthcare access, but also from more effective herd immunity effects enabled by their greater population density. Notably, the southern region’s intermediate incidence despite urban proximity suggests that population mobility patterns may outweigh density effects in some areas (34). Similarly, the northern rural region’s incidence patterns indicate that healthcare accessibility thresholds - rather than simple distance to services - best explain vaccination uptake and case detection rates. These findings move beyond simple incidence mapping to demonstrate how the interaction of geographic factors (border effects), infrastructure (healthcare distribution), and social determinants (mobility patterns) collectively shape disease landscapes. Future interventions should incorporate such multidimensional spatial analyses when targeting vaccination programs or surveillance efforts.

The study further explored the complex relationships between varicella incidence and various demographic characteristics, healthcare capacity, economic level, air pollutants and meteorological factors. Firstly, we found that there were strong correlations between various natural and social-ecomic factors. There were positive association between the children population and factors such as industrial output and energy consumption, while the sex ratio negatively correlated with household registration and population density. The VarV program’s success was negatively correlated with higher levels of air pollutants and acid rain frequency but positively associated with annual average temperature. Additionally, in the principal component analysis, we found that the incidence of varicella was significantly correlated with components child population ratio, healthcare level, VarV immunization program and air pollutants. Previous studies have also revealed that PM10 and population density can influence the association between wind velocity and varicella (29). The intricate interplay between these factors highlights the multifaceted nature of varicella epidemiology and underscores the need for a comprehensive approach to disease prevention and management.

Our analysis underscores the intricate relationship between demographic factors and varicella incidence. The principal components of child population ratio had a strong positive correlation with varicella incidence, echoing previous research by Tatina TT that identified demographic component was robustly associated with varicella incidence in Bulgaria (53). Upon examining the individual components of the child population ratio, we found that the household registration ratio had a significant negative association with varicella incidence. This correlation may stem from the fact that, although the varicella vaccine is not integrated into China’s national immunization schedule, Shanghai has adopted it into its local program. Consequently, children without local household registration, who are likely from other regions, might not receive varicella immunization promptly. Our findings suggest that population mobility significantly influences varicella transmission dynamics. Conversely, child population density showed a significant positive correlation, suggesting that denser areas might see higher incidence rates. This association could be intricately linked to various factors, including accessibility to medical resources, public awareness of preventive measures, promptness in seeking medical attention, and the level of urbanization within the region. Consistent with several previous studies (54, 55), while the varicella incidence and varicell cases among male children higher than females, this difference did not reach statistical significance. These results underscore the importance of considering demographic factors comprehensively when assessing the epidemiology of infectious diseases like varicella. The significant impact of household registration ratio and population density indicates that public health strategies could benefit from focusing on these aspects to control varicella spread.

While this study offers valuable insights, it is important to acknowledge potential limitations, such as reporting biases and data completeness. Personal identification information in the infectious disease reporting system may not fully match vaccination records, leading to information bias. Moreover, environmental and meteorological variables were derived from annual regional averages, which may limit the analysis of their association with varicella incidence. Finally, the influence of other unmeasured factors cannot be dismissed. Future studies should aim to strengthen surveillance systems and improve data collection to enhance the accuracy of findings. Despite these limitations, the study’s strengths include the use of a robust dataset and advanced statistical methods to analyze varicella incidence trends.

## 5 Conclusion

In conclusion, this study demonstrates the substantial impact of the two-dose VarV vaccination program in reducing varicella incidence in Jiading District, Shanghai. The findings underscore the importance of sustained vaccination efforts and targeted public health interventions in controlling varicella outbreaks. The child population ratio and the VarV program in Shanghai emerged as the two major determinants of varicella incidence in the region. By continuously monitoring and addressing the factors influencing varicella transmission, public health authorities can further diminish the disease burden and protect vulnerable populations. Overall, this study adds to the growing body of evidence supporting the effectiveness of varicella vaccination programs and emphasizes the need for comprehensive public health strategies to combat infectious diseases.

## Data Availability

The original contributions presented in the study are included in the article/Supplementary material, further inquiries can be directed to the corresponding authors.
