# Supplementary material for: Impact of natural and socio-economic factors on varicella incidence in children in Shanghai, 2013-2022
Source: Front Public Health. 2025 May 27;13:1565717. doi: 10.3389/fpubh.2025.1565717 (PMC12150853; doi:10.3389/fpubh.2025.1565717)
Supplement: Supplementary file 1 [file Supplementary_file_1.pdf]

Supplementary Material

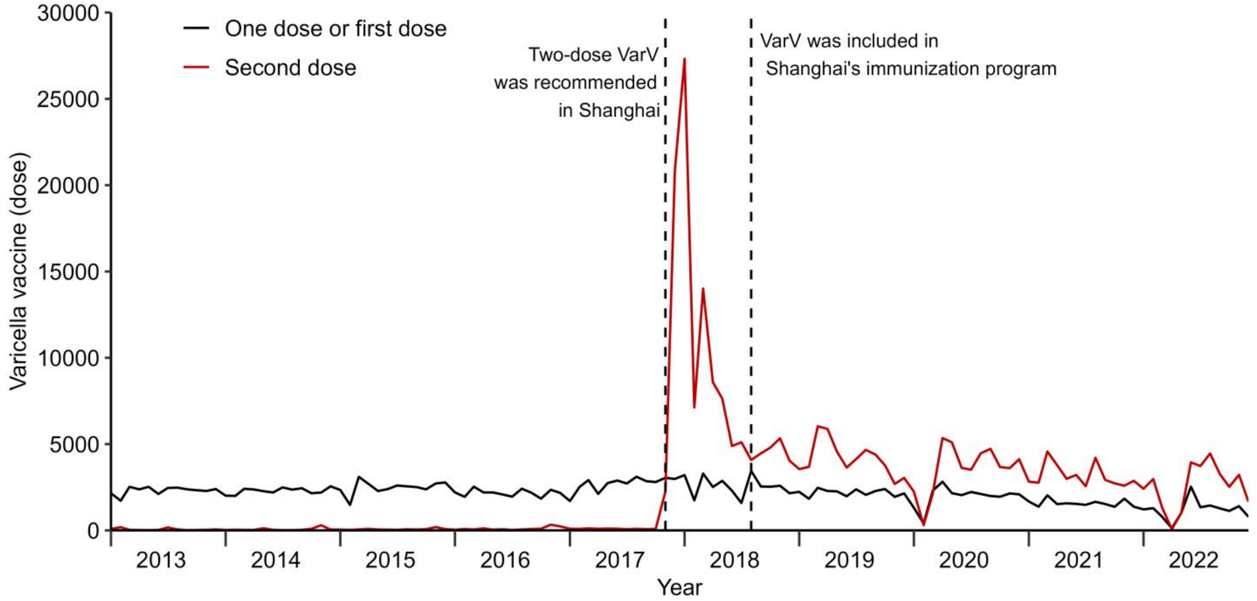

Supplementary Figure S1. Monthly distribution of varicella vaccine doses administered to children in Jiading District, 2013–2022.

Supplementary Table S1. Demographic characteristics of reported varicella cases in children in Jiading District, 2013-2022.

**Characteristics**	**Annual average incidence (per 100,000)**			**χ^2 or W value**	**p value**
	**All Study Period**	**One-dose VarV program Period**	**Two-dose VarV program Period**		
Sex				0.10*	0.755
Male	3803 (58.67)	2473 (58.52)	1330 (58.95)		
Female	2679 (41.33)	1753 (41.48)	926 (41.05)		
Age, years					
Median (IQR)	6 (3-11)	6 (4-10)	7 (3-12)	4,547,699**	0.002
Age group				264.10*	＜0.001
＜1 year	549 (8.47)	333 (7.88)	216 (9.57)		
1-3 years	1084 (16.72)	592 (14.01)	492 (21.81)		
4-6 years	1626 (25.08)	1292 (30.57)	334 (14.8)		
7-9 years	1159 (17.88)	806 (19.07)	353 (15.65)		
10-12 years	1019 (15.72)	622 (14.72)	397 (17.6)		
13-17 years	1045 (16.12)	581 (13.75)	464 (20.57)		
Occupation				75.33*	＜0.001
Scattered children	1514 (23.36)	901 (21.32)	613 (27.17)		
Nursery children	1572 (24.25)	1157 (27.38)	415 (18.4)		
Students	3321 (51.23)	2126 (50.31)	1195 (52.97)		
Others	75 (1.16)	42 (0.99)	33 (1.46)		
Region				37.25*	＜0.001
Central	1754 (27.06)	1129 (26.72)	625 (27.7)		
Western	1234 (19.04)	811 (19.19)	423 (18.75)		
Southern	2099 (32.38)	1290 (30.53)	809 (35.86)		
Northern	1395 (21.52)	996 (23.57)	399 (17.69)		
Vaccination status				498.33*	＜0.001
Unvaccinated	3486 (53.78)	2487 (58.85)	999 (44.28)		
One dose	2716 (41.9)	1723 (40.77)	993 (44.02)		
Two dose	280(4.32)	16(0.38)	264(11.7)		
Total number of cases	6482(100)	4226(65.2)	2256(34.8)		

*: χ^2 value by Chi-square test, **: U value by Mann-Whitney U test.

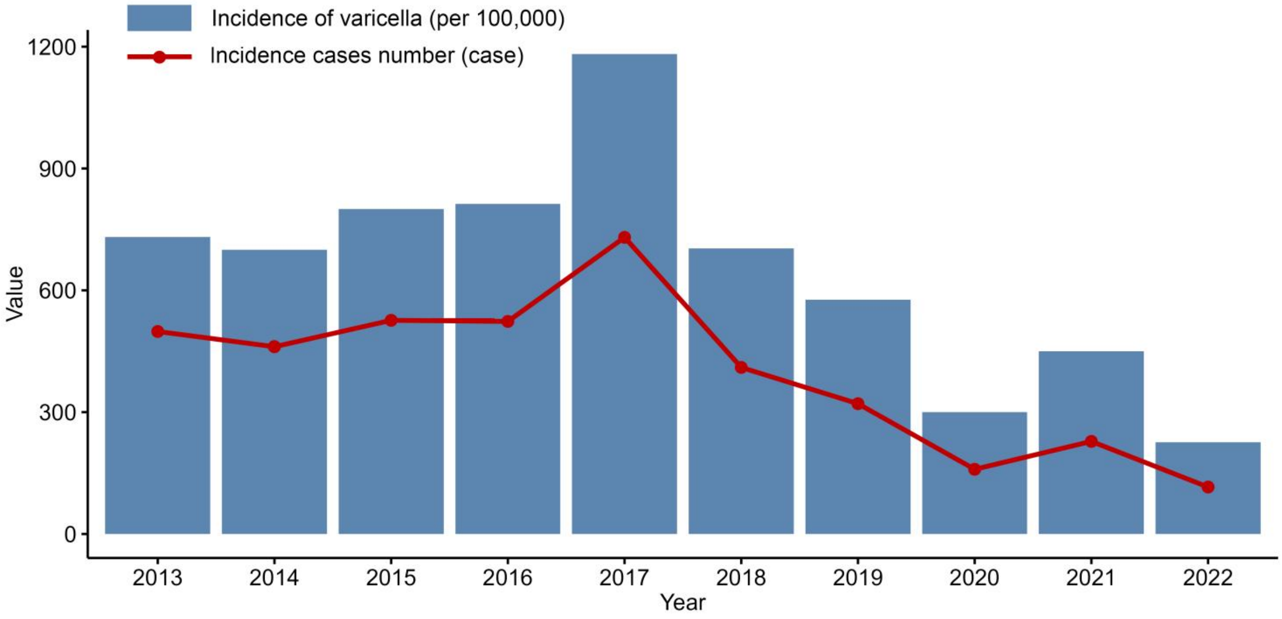

Supplementary Figure S2. Temporal distribution of incidence of varicella in children in Jiading District, 2013-2022.

Supplementary Table S2. The multiple regression analysis of incidence of varicella in children: VIF, and Partial R-squared Values for independent variables.

Variable	VIF	Partial R^2
Children population (100,000 persons)	3.47	<0.001
Sex ratio (male to female, %)	6.91	<0.001
Household registration ratio (registered to non-registered, %)	12.95	0.093
Child population density (person/square-kilometer)	9.24	0.045
VarV immunization program (0, 1)	21.82	0.007
Secondary and above comprehensive medical institutions (unit)	7.36	0.036
Total number of patients (person-time)	6.99	0.059
Number of health check-ups (person-time)	3.67	0.003
Completed investment in fixed assets (10,000 CNY)	4.54	0.007
Industrial output value above the set scale(10,000 CNY)	40.81	0.008
Profits of enterprises above the set scale (10,000 CNY)	5.37	<0.001
Comprehensive energy consumption (10,000 tons of standard coal)	29.38	0.003
Unit output energy consumption (tons of standard coal/10,000 CNY)	2.05	0.006
Particles with an aerodynamic diameter less than 10 μm (PM10, $\mu g/m^3$)	63.33	0.139
Sulfur dioxide (SO_2, $\mu g/m^3$)	22.48	0.017
Nitrogen dioxide (NO_2, $\mu g/m^3$)	45.43	0.157
Acid rain frequency (%)	26.72	0.008
Annual average temperature (℃)	14.53	0.239
Sunshine duration (h)	6.44	0.001
Relative humidity (%)	22.01	0.185

Multiple R-squared: 0.7451. Abbreviations: VIF, Variance inflation factors; Partial R^2, Partial R-squared; VarV, varicella vaccine.
